# Public Speaking

**Published:** 1843-04

**Authors:** 


*Public Speaking.*—One of the greatest errors committed by public speakers, when addressing large bodies of people, is speaking too fast. They forget that distance has the same effect upon sounds as it has upon architectural or other ornaments; it melts, as it were, the more minute parts into a confused mass. Elaborate and ornate passages in music cannot be appreciated by a moderately distant listener, while the bold and distinct slow movement can be felt and understood by him with ease.

